# “Economic exclusion and the health and wellbeing impacts of the oil industry in the Niger Delta region: a qualitative study of Ogoni experiences”

**DOI:** 10.1186/s12939-024-02248-7

**Published:** 2024-09-12

**Authors:** Augusta C. Nkem, Susan Devine, Daprim S. Ogaji, Stephanie M. Topp

**Affiliations:** 1 College of Public Health Medical and Veterinary Sciences, James Cook University, James Cook Drive, Townsville, QLD 4811 Australia; 2 Africa Centre of Excellence in Public Health & Toxicological Research (ACE-PUTOR), University of Port Harcourt, Choba, Port-Harcourt City, Rivers State Nigeria

**Keywords:** Oil industry, Social exclusion, Economic impacts, System Injustice

## Abstract

**Background:**

When managed effectively, oil wealth can drive economic development and improve wellbeing. Conversely, as has been the experience in Ogoniland in the Niger Delta, the presence of the oil industry can lead to environmental degradation and social and economic vulnerabilities with negative impacts on health and wellbeing. Few studies have explored the experiences and perceptions of these impacts among residents of host communities themselves.

**Methods:**

This qualitative study explored Ogoni residents’ experiences and perceptions of oil-industry related economic exclusion and associated impacts on health and wellbeing. In-depth interviews with 22 participants from four Local Government Areas (LGAs) were analyzed with open (inductive) coding. Guided by constructivist philosophy, interpretation of emerging themes was informed by the concept of social exclusion which recognizes that multi-dimensional processes can deprive individuals or groups of resources, rights, goods, and services, thus limiting broader societal participation.

**Results:**

Findings highlight the exclusionary impacts of the oil industry at the intersection of i) damaged livelihoods and family income, that increased economic vulnerability and reduced participants’ ability to meet basic needs including ability to pay for healthcare; ii) lack of progress on environmental remediation and non-transparent benefit sharing schemes that exacerbate economic displacement and contribute to ongoing exposures to air and water pollution; and iii) insufficient of investment in economic development and essential health services, limiting employment opportunities and ability to access adequate healthcare.

**Conclusion:**

Addressing these issues requires integrated policy interventions focusing on equitable resource distribution, environmental restoration, and inclusive economic development to promote sustainable community resilience.

**Supplementary Information:**

The online version contains supplementary material available at 10.1186/s12939-024-02248-7.

## Background

When managed effectively, oil wealth can drive economic development and improve overall wellbeing elevating living standards through funding massive infrastructure projects, healthcare advancements, and social welfare programs that help reduce poverty and improve health outcomes [1]. Oil wealth managed through sovereign funds, in particular, has contributed to raising and sustaining high standards of living and robust social services [2]. However, negative impacts of oil production on economic and health and wellbeing outcomes are also well documented. Mismanagement and over-reliance on oil exports in some countries have triggered economic crises, contributed to social unrest, and even deepened poverty [3]. Pollution and environmental degradation caused by unchecked or poorly regulated downstream and upstream processes have disrupted ecosystems and damaged food systems and livelihoods [4], and been linked to health issues such as respiratory and cardiovascular diseases [5]. In a number of oil producing states, production has been linked to conflict and instability and entrenched political power and social inequalities [6–8].

In Nigeria, oil accounts for approximately 85% of the country’s total revenue. Since the 1970s, however, contestation over the distribution of oil wealth has led to a complex array of wealth distribution mechanisms as local, state and federal governments and host communities themselves have sought to benefit from oil revenue [9]. In 1971 the Nigerian National Oil Corporation (NNOC) was established to manage state involvement in oil production. This entity merged with the Ministry of Petroleum Resources in 1976 to form the Nigerian National Petroleum Corporation (NNPC), responsible among other tasks, for equitable revenue distribution. In 1989 the Oil Mineral Producing Areas Development Commission (OMPADEC) was created to manage the allocation (at the time) of 1.5% of oil revenue to oil-producing areas. By 1999 a 13% Derivation Principle was enshrined in the Nigerian Constitution, mandating that 13% of oil revenue be allocated to the nine oil-producing states to address environmental and infrastructural impacts in the region. The Niger Delta Development Commission (NDDC) was established in 2000 (replacing OMPADEC) to administer that 13% derivation and bolster regional development. Two national-level redistribution mechanisms were also established in 2005, the Excess Crude Account, (ECA) designed to stabilize revenue allocation amidst oil price volatility; and in 2011, the Nigerian Sovereign Investment Authority (NSIA), designed to manage a sovereign wealth fund for long-term national economic stability and infrastructure development. While exact revenues are difficult to track, one report estimated that the oil-producing states were allocated N6.588 trillion ($4.35 billion) between 2009 and 2019 [10]. Rivers State, with an estimated population of 7.5 million and among the top three oil-producing states in Nigeria was allocated approximately ₦1.056 billion ($41.72 million) [11, 12].

**Fig. 1:**
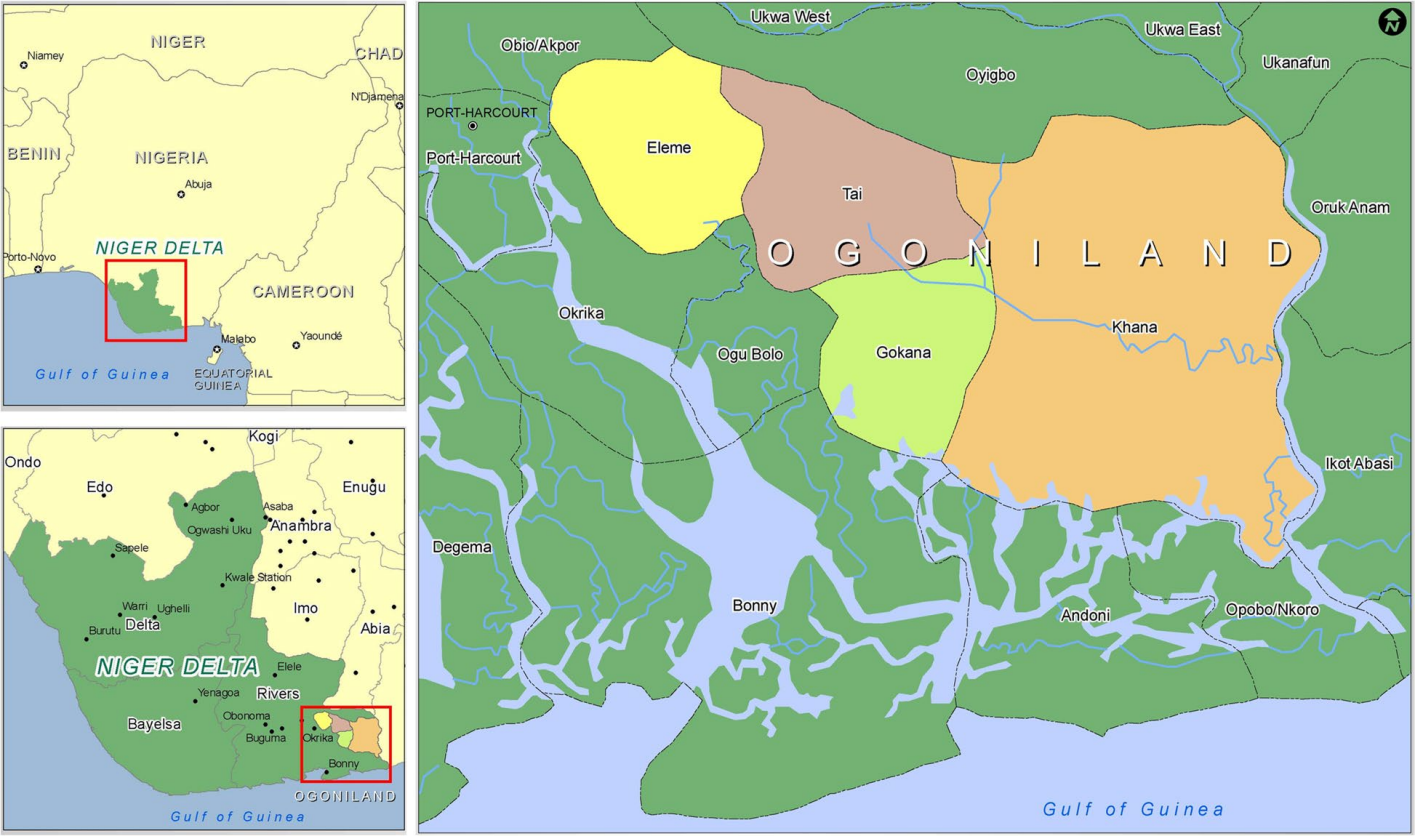
Niger Delta and Ogoni Region. Source: [15]

Despite the significant oil wealth generated in the Niger Delta, economic and human development in the region has been slow, and environmental impacts profound. In the Ogoni region of Rivers State, a traditional kingdom covering approximately 1000 square kilometres, (Fig. 1) exploration and exploitation of oil resources started in the 1950s under the Shell Petroleum Development Company (SPDC), the largest oil producer in the country [13]. Operations in the region came to an abrupt halt in the early 1990s, however, amidst demands from Ogoni leaders for greater political and economic resource control, representation and environmental protection. Tensions over initially peaceful protests and Ogoni demands, led to escalation and violent clashes between state and federal government forces and local communities in the early 1990s, and the 1995 arrest and execution of nine Ogoni leaders [14]. Following the arrests, the Nigerian Petroleum Development Company (NPDC) assumed control of SPDC assets in Ogoniland (including five non-producing fields and a network of approximately 100 wells and the related infrastructure).

Figure 1 illustrates the geographical location of the NDR, with a focus on the Ogoni Region within the Rivers State highlighting the significant areas of impact. Source: Adapted from [15].

The environmental consequences of oil activities under SPDC and NPDC control in Ogoniland have been severe. In 2011 the United Nations Environment Programme (UNEP) conducted a landmark environmental assessment of Ogoniland [16] and documented catastrophic environmental impacts, including over 2,976 separate oil spills, equivalent to two million barrels of oil, between 1976 and 1991 alone. More recent data from Nigeria’s National Oil Spill Detection and Response Agencies (NOSDRA) indicates the on going nature of that challenge with 535 spills reported by SPDC across all sites (including Ogoniland and other regions) in 2023 alone [17]. Oil spills contaminate surface water, ground water, ambient air and crops with hydrocarbons, including carcinogens that bio-accumulate in some food crops [18]. Oil spills have been estimated to have led to a 60% reduction in household food security as well as a reduction in the ascorbic acid content of vegetables by as much as 36% and the crude protein content of cassava by 40% [19]. Despite being rich in oil, gas and many other natural resources, a 2006 the United Nations Development Program report noted the that a majority of the people in the Niger Delta live on the margins [20].

The juxtaposition of significant resource wealth set against the catastrophic environmental and developmental impacts in Ogoniland and Niger Delta more broadly has been characterised by some as a ‘resource curse,’ [21, 22], where natural resources cause social and economic exclusion rather than prosperity. Over the past twenty years, research on the oil industry’s impacts has highlighted the influence of social determinants on health and well-being [3, 23, 24]. Studies have documented the contamination of water sources and agricultural land that has diminished productivity and brought economic losses to local communities [25–27]. Researchers such as Wiwa [28], Brino [29] and Habiba [30] have described the unequal distribution of oil revenue as a factor contributing to low income among some residents of oil communities in Nigeria. Additionally, studies have documented the compromised health and well-being of Ogoni residents, which has exacerbated poverty and social inequality[31]. Some research has examined economic dimensions like regional revenue generation and compensation schemes[25, 32] however, relatively little work has focused on residents’ own experiences or perceptions.

This study set out to examine Ogoni residents’ perceptions and experiences of the economic impacts of oil industry activities and their related health and wellbeing impacts, responding to two questions: 1) *how do Ogoni indigenes perceive and experience the economic impacts of the oil industry?*; and 2). *in what ways are these economic impacts understood to relate to individual and collective health and wellbeing*? Understanding these experiences can highlight the voices of those most impacted, and contribute to health and environmental justice discourse and advocacy. Set within a broader project exploring the experiences of oil-industry-related social exclusion in Ogoni land, this study thus aims to explore Ogoni residents’ experiences of economic impacts and related health and wellbeing impacts related to the presence of oil industry in the region.

## Methods

### Design

**Fig. 2:**
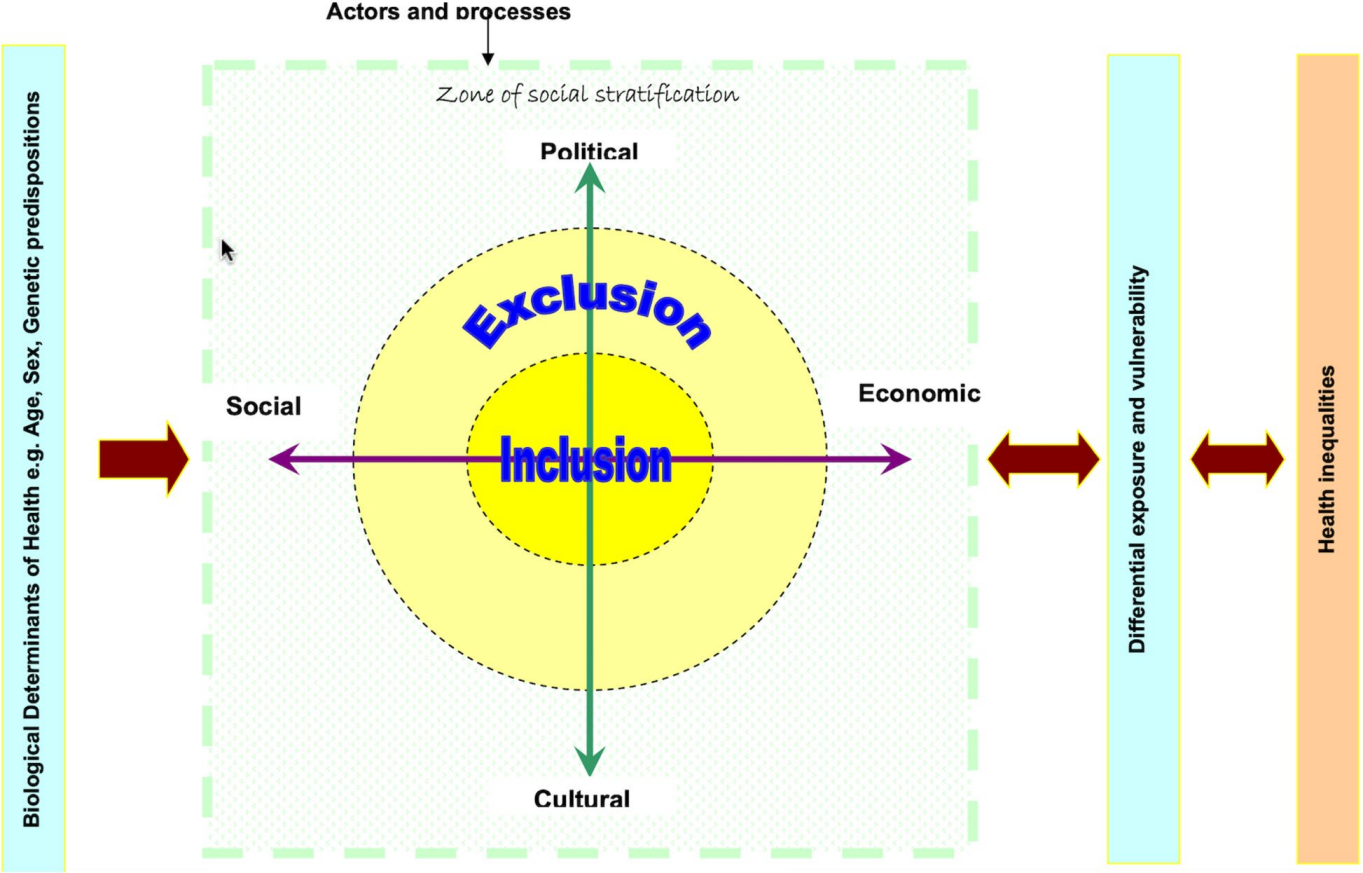
Social Exclusion Knowledge Network (SEKN) framework [38]

The qualitative study was grounded in the principles of constructivist philosophy. This approach is appropriate for exploring the experiences and perceptions of participants because it allows for an in-depth understanding of their subjective realities and social contexts and recognizes that knowledge is co-constructed through interactions, providing rich, nuanced insights into the participants’ lived experiences.[33]. As a concept, social exclusion (which includes economic exclusion) refers to the systematic denial of resources, rights, goods, and services, and the inability to participate in the normal relationships and activities available to most people in a society, which critically undermines both individual and community wellbeing [34–36]. Diverse frameworks have been formulated to investigate social exclusion in different contexts [37], with one notable example being the World Health Organization's (WHO) Social Exclusion Knowledge Network (SEKN) framework (Fig. 2) [38]. SEKN adopts a relational perspective, conceptualizing social exclusion as a dynamic, multi-dimensional process rooted in unequal relationships [38]. This framework considers the impact of agents across four dimensions—political, social, economic, and cultural— which lead to outcomes that can either benefit or disadvantage the individuals within the social system. Exclusionary processes operate within social systems, spanning families and household contexts through to national and global domains. These processes are shaped not only by social dynamics but also by biological factors such as age, gender, and genetic predisposition. Furthermore, interactions among the four dimensions of social exclusion—political, social, economic, and cultural—are influenced by the systems of social stratification that shape education, occupation, income, and wealth, and determine access to resources and ability to mitigate health-related risks. Lower levels of stratification are likely to increase vulnerability and perpetuate inequalities, ultimately impacting health and well-being [38].

Figure 2 presents the SEKN framework derived from [38] WHO-led research.

In the larger project of which this study forms one part, the SEKN framework serves as a sensitising concept, guiding the exploration of the four primary dimensions associated with social exclusion: economic, cultural, social, and political. This study focuses specifically on the economic dimension of social exclusion, exploring the unique economic dimensions of the oil industry’s impacts on health and wellbeing in the region. This study offers detailed insights into the wider implications of oil industry activities on the people of Ogoni, particularly within the economic domain.

### Setting

This study was conducted in the Ogoni region of Rivers State, Nigeria, which is home to 832,000 individuals out of the State’s 7.5 million residents, [39]. The region also hosts numerous oil companies, including Shell which has up to 50 facilities in NDR with more than 1700 companies, and extracts approximately 100 million barrels of crude oil annually [40, 41]. This region has experienced a high frequency of oil spills and other incidents [42, 43].

### Sampling, recruitment and data collection

A stratified purposive sampling approach combined with snowball sampling, was used to ensure the representation of diverse community members within the project’s resource limits, and to ensure insights were obtained from the extensive experiences of Ogoni residents who have lived in the oil extraction region for over four decades. The main criteria for stratification was residency across the four different local government areas (Eleme, Gokana, Khana, and Bori), type of employment (informal, local government officials, oil industry workers and land owners) and gender. Individuals had to be adults aged 18 years to participate.

Twenty-two participants who resided in the four Local Government Areas (LGAs) within the Ogoni region, specifically Eleme (*n* = 6), Gokana (*n* = 4), Khana (*n* = 6), and Bori (*n* = 6) participated in the interviews. The participants were comprised of 10 females and 12 males with 13 engaged in formal employment. Employees hold positions in government, health, farming, fishing, private businesses and the oil industry.

Various methods were used to recruit participants, starting with the dissemination of an information sheet distributed across various social media platforms and community groups. Invitations were extended to contact either the primary investigator (AN) or the research assistant via WhatsApp, for inquiries or participation in interviews. The first author’s (AN) professional networks, cultivated through active engagement in social and community groups, that connect via Facebook and WhatsApp within the region, were also used. These networks facilitated the dissemination of study information through community forums, including market women and youth groups. Prospective participants expressing interest in the study were provided with comprehensive briefings either in-person or via WhatsApp, affording them the opportunity to seek clarification before consenting to participate in either face-to-face or virtual interviews. Concurrently, a research assistant engaged in face-to-face outreach activities at public venues such as markets to disseminate study information, answer questions, and coordinate interviews with interested individuals. Snowball recruitment complemented these efforts, as community members referred to or recommended the research team to other potential participants. Recruitment was halted when preliminary analysis indicated data saturation had been reached. Despite initial plans for a larger sample, an evolving security situation (further detail in Strengths and Limitations below) also curtailed continued data collection. Data were collected from April 2022 to November 2022.

Prior to commencing the study, a research assistant (with a Master’s degree in public health from the University of Port Harcourt, Nigeria) was recruited and received extensive training on the objectives of the study and interview-based data collection methodology. This training aimed to equip the researcher with the necessary skills, achieve consistency in data collection and effectively follow the ethical guidelines. Data was collected through semi-structured, in-depth interviews. A pre-tested interview guide was used to facilitate discussions with participants about their experiences of social exclusion in relation to the oil industry. The question guide was developed with reference to the four domains of social exclusion – social, economic, cultural, and political—with the findings reported here focusing primarily on the economic domain. Initial guides were refined and adapted for clarity following several rounds of piloting and are available in Appendix 1.

Interview questions were open-ended, allowing the participants to freely express themselves. The interviews were conducted in either NDR pidgin or English, based on the participants’ preferences, and full engagement in the conversation was encouraged. Participants who agreed to be interviewed (via WhatsApp or in person) were required to provide written informed consent prior to the session, with the assurance that they could withdraw at any time without any negative consequences. A total of 22 interviews were conducted, comprising 19 face-to-face sessions and three via WhatsApp, based on the participants’ preferences. To accommodate participants who were busy with marketplace activities, interviews were often conducted in quieter locations away from the market. Interviews with retired older adults were conducted at home upon request. The interview durations ranged from 20 to 50 min and were recorded with the participants’ permission. Following the interviews, the first author (AN) and research assistant conducted debriefing sessions and documented these conversations with memos.

### Data analysis

A transcription service translated and transcribed the Pidgin audio recordings verbatim in English. After the transcription, the first author (AN) reviewed the transcripts along with the original recordings to ensure accuracy. The interview transcripts and debriefing notes from the meetings between the research assistant and primary author following each interview were imported into NVivo QSR™ for analysis. An open coding process was used with research team members individually reviewing transcripts to develop coding ideas that contributed to the establishment of an initial codebook (AN, ST, and SD). The main focus of the coding was on the descriptions and experiences related to experiences of economic exclusion and their health and wellbeing impacts on Ogoni residents. In the continuing rounds of coding led by AN, codes were categorised, grouped under umbrella codes, and organised into themes. Subsets of codes addressing the economic impacts were prioritised. The first author (AN) developed a summary for each code related to economic impacts and engaged in extensive discussions with the co-authors to clarify their meanings, interpretations, and interconnections. Member checks with original participants were not sought due to the pragmatic and security related challenges of re-tracing community-level interviewees post-analysis (during a period of post-election unrest and due to concerns for the personal safety of the research assistant). Rather the first and third author (DO), as well as the research assistant with long experience living and working in the region each reflected on gaps and interpretations of the data. The process was additionally informed by constant comparative techniques, that enabled a flow examination of the data to discern patterns, differences, and themes of economic impacts. The use of the constant comparative analysis helped ensure thorough examination of the details within the coded data, enhancing the understanding and depth of the economic impact aspects being studied.

### Reflexivity and standpoint

Our authorship group is composed of individuals with diverse and complementary backgrounds, each contributing unique perspectives and expertise to the research. AN is a Nigerian PhD candidate with a background in the oil industry and extensive experience living and working in the Niger Delta region for 12 years. AN’s firsthand knowledge of the industry and local context provides valuable insights into the community’s lived experiences and the impact of the oil sector. DO, a Nigerian academic based in Port Harcourt with specialized expertise in the public health impacts of the oil industry. DO’s academic and professional experience offers a critical understanding of the health challenges faced by communities in oil-producing areas. SD’s deep understanding of public health related social determinants and SMT’s extensive understanding of global health and policy ensured the research was situated not only locally but within a broader international context. This combination of local knowledge and understanding, and broader global insights allowed for a more insightful understanding of the issues raised by participants.

## Results

### How Ogoni indigenes perceive & experience the economic impacts of the oil industry

Participants described three major forms of economic exclusion with implications for health and wellbeing. First, the erosion of traditional income sources and associated financial vulnerability which contributed to the inability to pay for healthcare. Second, weak environmental remediation and non-transparent wealth-distribution schemes that lead to ongoing health impacts and high levels of stress. Third, lack of investment in economic development and essential health services, limiting employment opportunities and ability to access adequate healthcare. Below we describe each theme more fully, and in a second section outline their intersection with health and welling outcomes.

Theme 1: The erosion of traditional income sources and associated financial vulnerability.

Participants described the financial impacts of reduced agricultural productivity and the disruption of fishing activities caused by the oil industry. These impacts have arisen because of land claims associated with the Land Use Acts of 1978, and the subsequent environmental degradation associated with oil industry activities. The Nigerian Land Use Act of 1978 centralized land ownership in the hands of the state. Although the Act was claimed to enable development and eliminate the complexities and inequalities associated with pre-existing land tenure systems, it had dire consequences for Ogoni residents. Communities whose livelihoods were deeply intertwined with land through agriculture, fishing, and other traditional industries experienced rapid economic impacts, as access to land and land quality was degraded.

*“A very vast portion of our land is taken. Before now, we had good land and rivers. And when pollution came, the whole thing got damaged beyond. We no longer drink good water, we no longer have fish, and we no longer have farms.” (Landlord, Int 17).*

“*In Ogoni land, oil wells have taken over the entire place. The presence of oil wells in the land has made it impossible to farm.” (Industry Staff, Int 3)*.

As the quote above reveals, the redirection of land for industrial and oil production purposes was worsened by the continuous environmental impact of oil spills and flaring. Interviews included multiple accounts of the livelihood challenges including poor crop yields, reduced fish stocks, and poisoned water. Participants described disruption of marine ecosystems and aquatic habitats; and reductions in periwinkle, a culturally significant food source for Ogoni as well as an important source of income. Several older participants recalled a time before the oil industry arrived when they had a bountiful catch of fish, emphasising that the decline in fish availability, was caused by water pollution as noted below:

*“Back in those days before oil extraction, people could easily go to the river for fishing and would come back with fish or crabs.” (Fisherwoman, Int 16)*

According to one participant ground instability caused by the oil industry affected soil quality and fertility, and also threatened the sustainability of agricultural practices.

*“If you put mortar on the ground to pound yam such as during Ogoni peoples’ day; when you pound, it looks as if the earth would collapse. You will be hearing echoes as if the earth is collapsing and sinking.” (Community Resident, Int 20).*

Participants also explained the compounding impacts of these losses on livelihoods and household income, as they had too little to sell or trade and lost a key means of economic participation and power.

*“It affects our occupation, it affects financially because if there is no better production of our crops, we cannot have money as usual.” (Elder, Int 10)*.

*“It’s all because of no job and there is no economic power. In the past, people would gather by the river and engage in fishing activities, bringing along crabs and other things.” (Fisherwoman Int, 16).*

Theme 2: Weak environmental remediation and non-transparent wealth-distribution schemes.

Despite acknowledging the existence of environmental remediation schemes many participants were emphatic that little had been done to effectively mitigate either soil or water pollution.

*“To clean the entire environment [is required] so that the farmland- crops can grow better. But nothing has been happening ‘till now.” (Self-employed, Int 1)*.

Several participants characterised their experience of government inaction as indicative of a general lack of accountability. In the quote below a community farmer expressed deep-seated frustration and distrust towards the government and its officials. The participant recounts an instance where the Minister of the Environment visited Ogoniland to check the progress of new water infrastructure, and the clean-up efforts associated with the Hydrocarbon Pollution Remediation Project (HYPREP or ‘Ogoni Clean up’) and reflected a sense of betrayal and abandonment evidence too in other accounts.

*“There are some things I witnessed and still can’t understand. How can a Minister of the Environment come to check the progress of work done and remain in his vehicle? And it is his responsibility to check what they are doing. Take the water infrastructure here in Bori, for instance, and they have not done anything so far. Nothing has been done for about 4 to 5 years since they claimed to have started the Ogoni clean-up. If you want to come and see, you are free to come and see for yourself that nothing has been done until now. Yet they claim they are in phase 3 to 4 of the clean-up exercise, yet nothing has been done. These are the people responsible.” (Community Farmer, Int 8).*

Related to but distinct from the issue of environmental remediation, participants also described their experiences of the inadequacy of benefit-sharing schemes. Although few actually mentioned specific schemes (for example the 13% derivation funds) participants expressed the perception that oil revenue was financially benefitting national and state level political elites and those with direct ties to oil companies, while local communities were left to face environmental degradation without adequate compensation or improvement in living standards.

*“Despite being the owners of the oil, the local population derives no benefits from its extraction; instead, the profits are enjoyed in other parts of the country like Abuja and Port Harcourt.” (Self-employed, Int 1).*

There were also some respondents who believed that oil companies themselves were not sharing wealth appropriately:

“*The companies are not compensating us adequately. I say this because I am very close with most indigenes and there had been no compensation and I have not seen any good life or anything good come from any of those companies.” (Industry staff, Int 4).*

These perceptions were compounded by experiences of ad hoc schemes by oil companies such as distribution of small monetary gifts, or annual gifts of food or animals to residents by the SPDC. Others described oil companies negotiating with individual community leaders or factions within communities.

*“They often offer them food items like rice and to each community, a cow as well as monetary gifts for the Christmas celebrations.” (Industry staff, Int 3)*.

Most participants described these ‘gifts’ and other forms of compensation as either insufficient, or lacking appropriate consultation and transparency, with several noting they contributed to wealth inequity and underlying disputes within and between communities.

Theme 3: Lack of investment in economic development or essential services.

The third theme related to a lack of investment (by government or industry stakeholders) in economic development and essential services, and a concurrent lack of employment prospects. Challenges accessing healthcare and education were mentioned by many participants. A number of participants described a sense of being abandoned by both the government and the oil companies, with one participant below, describing the situation as needing to rely solely on their faith.

*“No good healthcare, no health. We are living at the mercy of God.” (Self-employed, Int 1).*

In one case, a participant acknowledged a government sponsored scheme that had provided medications for hypertension – a common condition in the community. However, this had since ceased, and experiences of systemic failures in basic health services left a number of participants feeling vulnerable and unsupported.

*"The government used to send drugs, and the oil company used to send drugs for us to calm this stress of a thing we are talking about. But for how many years now, for about four years, we have not been seeing anything like that.” (Community Resident, Int 9).*

In part because of the loss of traditional livelihoods and the need to find alternative sources of income, participants noted education was important to finding alternative employment. Some participants described a perceived lack of government investment in education for youths in particular.

*“The youths lack the opportunity to go to school.” (Community Resident, Int 15).*

The link between limited access to health and education broader economic inclusion found expression in participants reflections on the recruitment practices of oil companies who were observed to prefer those with specialised skills, specific qualifications, or technical expertise. At the same time, however, several participants noted that oil firms such as SPDC conducted their major recruitment from urban centers rather than the LGAs that were host to the oil operations. Both the mismatch in skills and qualifications, and the recruitment practices were perceived as exclusionary.

*“The presence of an oil industry here is expected to provide employment opportunities, however, we have not been successful in obtaining employment.” (Community Resident, Int 15).*

*“SPDC do their employment in Lagos, they do not consider the Ogoni people.” (Self-employed, Int 1).*

*"We will not see youths who are graduates for employment.” (Community Resident, Int 20).*

Participants noted the compounding effect of lack of employment and localised inflation created by the influx of well-paid non-resident workers whose relatively high salaries enabled them to pay more for goods and services, driving up prices for locals too.

*“They are not providing job opportunities, and everything is expensive.” (Community Resident, Int 15).*

### How economic impacts are understood to relate to individual and collective health and wellbeing

Resident’s experiences of the health and wellbeing impacts from these intersecting forms of economic exclusion were profound. Inadequate progress on environmental remediation were viewed by most participants as having significant negative impacts on community health and wellbeing. Despite widespread acknowledgement by government and industry of the negative environmental impacts and high-profile remediation schemes, participants described experiencing of delays in providing clean water in particular, linking their chronic exposure to harmful substances in the water, to respiratory and other systemic health issues.

*“The government is supposed to provide clean water because the lack thereof is causing so many illnesses […]. The government doesn’t care about us.” (Community Farmer, Int 8).*

*“Because when you take in the water, it damages your lungs and your health. You find out you cannot see a young person up to 60 to 70 because the water we are taking is one of the basic things that affect our health.” (Community Resident, Int 18).*

While some were able to avoid the health risks of drinking contaminated water by purchasing clean (bottled) water, this was unaffordable for many. This economic inequality exacerbated both health and wealth disparities, with poorer families suffering worse health due to their inability to afford bottled water.

*“Let me say drink Cway [bottled] water because we believe that one is somehow clean. How many families will be able to afford Cway water?” (Community Fisherwoman, Int 16).*

Prolonged exposure to explosions associated with gas flaring, were also linked by participants to health impacts such as hypertension and acute stress, with one participant also noting the inconsistent nature of support for managing this condition.

*“Explosion from the oil industry was generating noise and vibration—the whole community […]. It might affect other people because it affected me. It made me develop hypertension. My heart sometimes pounds, which has affected me since then. (Community Resident, Int 9)*

In this context of widespread environmental hazards, lack of income was described as compounding poor health and wellbeing outcomes, hindering their ability to meet basic needs like food security, or afford healthcare.

*“The cost of living is very high and coupled with a lack of job opportunities. So, for people who are not working, it means they don’t have money. So how can they even take care of their health when they have been affected by these gassy chemicals coming from these oil industries? Even when they have run a test, and they know the problem they’re going through, they cannot afford even to get proper medication.” (Community Resident, Int 15).*

*“People are getting sick because of no money. We don’t have a medical centre again. Maybe [in some places] you go to the hospital, and they treat you for free. There is nothing like that in Ogoni land. If you don’t have money if you fall sick, then you don’t have money, you will die. Because once you rush your person to the hospital, what they will request first is money, so because of that, people are dying. “ (Community Resident, Int 12).*

As outlined in the quotes above, both physical access to, and subsequent affordability of healthcare were challenging and participants linked these issues to illnesses going untreated, deteriorating health, increased physical suffering, and potentially long-term disabilities. Lack of government investment in availability of free healthcare services, in spite of the regional oil wealth, was described as exclusionary, with participants noting the necessity of upfront payments for healthcare as a cause of preventable deaths.

## Discussion

Social exclusion, broadly defined, perpetuates cycles of disadvantage and deep-rooted inequalities, including income and wealth disparities, and the ability to mitigate health risks [38]. Individuals or communities subjected to economic forms of social exclusion often experience food insecurity and face barriers in accessing healthcare, further exacerbating disparities in health and wellbeing. This qualitative study aimed to investigate experiences and perceptions of economic exclusion and their health and wellbeing impacts of the oil industry among residents of Ogoniland. It builds on existing literature by centering the voices of indigenous residents, and improves understanding of the multi-layered and temporal impacts of oil industry at the intersection of economic participation and health equity [44–46].

Our findings highlight residents’ experiences and perceptions of economic exclusion linked to loss of access to traditional farming land and widespread environmental damage that limit fishing and agricultural activity[41]. Historical expropriation of traditional lands in the Niger Delta began in the 1950s and was aided by the Nigerian Petroleum Act of the 1969s, which placed all land and resources under federal control. The 1978 Land Use Act subsequently authorised the government to appropriate land for public purposes, including oil exploration. Since that time, cumulative evidence has demonstrated extensive environmental damage and widespread pollution of air, water and soil across the Niger Delta and in Ogoniland in particular [16, 47, 48]. The 2011 UNEP report which focussed specifically on Ogoniland, identified severe environmental contamination, with oil from multiple spills penetrating the soil up to five meters deep, rendering much land infertile and unproductive for agriculture. The report also noted extensive water contamination, including high levels of hydrocarbons in surface water and sediment. Benzene, a known carcinogen, was found in water samples at levels 900 times above the WHO guidelines for drinking water.

Loss of livelihoods and income were linked by participants to the ongoing environmental impacts of oil extraction, but also to ineffective remediation and environmental clean-up efforts. Indeed, use of international courts to hold oil companies accountable for environmental damage has been a path of last resort for several frustrated host communities [48]. Following recommendations of the 2011 UNEP report, the Nigerian government established the Hydrocarbon Pollution Remediation Project (HYPREP) in 2016 to oversee Ogoniland clean-up and to catalyse community engagement through initiatives such as youth training programs. This was funded with $1 billion from Shell and other oil companies. Yet it was 2019 before HYPREP started implementation, and aligning with findings from this study, independent reports have noted slow progress, mismanagement, and inadequate community involvement [49].

Participants perceived their exposure to contaminated air, soil and water as an important factor in prevalent ill health, shorter life expectancy. Such experiences align with previous research demonstrating links between the Niger Delta contamination of water sources and crops with hydrocarbons, including known carcinogens, and heightened prevalence of kidney disease, cancers, and respiratory conditions in the region [18, 50]. One recent study demonstrated that people living in gas-flaring host communities in the Niger Delta have 1.75 times the risk of hypertension compared to those in communities without oil exploration activities [51]. Food insecurity resulting from reduced agricultural productivity has also been linked to a 24% increase in the prevalence of childhood malnutrition in the region [18]. Inyang and Simon [31], reported significant psychological distress among Niger Delta residents, attributed to ongoing environmental devastation and lack of economic opportunities. In Akwa Ibom state, Nriagu and Udofia [50] described high levels of disease symptoms and environmental distress (worry, annoyance and intolerance) associated with oil pollution.

Study findings also indicated the exclusionary impacts of both hard to reach, and expensive services, with participants mentioning lack of access to both healthcare and education. Contrary to the accounts of some participants in this study, positive impacts form (state) government education and scholarship schemes have been documented in one of the few studies evaluating the impact of government and oil company social policy interventions in Ogoniland [52]. 

Yet, the same study concluded that government and oil company healthcare interventions had had no positive impact on the Ogoni population and identified household income as the major determinant of health status in the region. Ogoniland has 71 primary healthcare facilities spread across its four LGAs (Eleme, Gokana, Khana, and Tai) although fewer than half may be operational at any given time [52]. Utilization of healthcare facilities has also been documented to be very low with major contributing factors including the distance from home, poor quality of services, delay in service delivery, lack of adequate medical personnel and, in line with findings in this study, the high cost of services [52, 53]. As demonstrated in our findings and elsewhere, limited reach or impact of such services intersects with high healthcare needs of local populations to significantly contribute to health inequity [52].

Participants in this study perceived a lack of state government investment in economic and human development infrastructure and services, which, combined with the loss of traditional livelihoods, compounded their economic exclusion. High unemployment rates, particularly among youth in Rivers State (27.9%), were significantly higher than Nigeria’s national unemployment rate of 21.1%, reflecting a rising trend in youth unemployment across the Niger Delta since 2016 [54]. Even those employed within the oil industry faced a highly casualized workforce, characterized by lower pay and less job security in both Nigerian and internationally owned companies. This economic vulnerability, resulting in reduced household income, led to difficulties in accessing or affording healthcare due to travel costs and out-of-pocket charges.

Participants also highlighted weak or absent corporate and political accountability by oil companies and various levels of government, which left their communities marginalized. Institutional arrangements for benefit-sharing from oil revenue have a long and complicated history, influenced by political priorities and attempts to curtail corruption and regulatory capture [55]. In 2021 the passing of the Petroleum Industry Act (PIA) brought another change with a new regulatory and governance architecture and the establishment of an additional benefit sharing mechanism – the Host Community Development Trust Fund (HCDTF). The purpose of the HCDTF includes providing direct social and economic benefits to host communities, [47] but early analysis of the act and the Fund have been equivocal about its prospects for reforming long standing issues around corruption and regulatory capture [56]. Despite the nominal presence of various benefit-sharing schemes over many decades, participants in this study were emphatic that these scheme had had little impact on their own economic opportunities. They expressed confusion about how the region’s substantial wealth resulted in so little tangible economic, social, or human development. Popay et al. [38] described social exclusion as a multi-faceted concept that cuts across economic political, social, and cultural aspects of community or individual experience and results in health and wellbeing inequalities. The SEKN framework [38] posits that exclusion occurs through multi-dimensional processes that deprive individuals or groups of resources, rights, goods, and services, thereby limiting their participation in normal relationships and activities available to the majority of people in a society. While much has been written about the impacts of the oil industry, a range of non-empirical sources (e.g., commentary, desk review, or opinion [39, 57–61] may make the evidence on social exclusion in Nigeria, and Africa more broadly, appear larger than it is. A recent review exploring empirical literature on the oil industry impacts in Africa, across the four major dimensions of social exclusion reported only a limited body of qualitative work on the lived experiences of residents in affected settings [62]. This study thus adds to the knowledge base by evidencing Ogoni residents’ experiences, highlighting the exclusionary impacts of the oil industry at the intersection of i) damaged livelihoods and family income, ii) limited reach or efficacy of environmental remediation, or government or industry investment in critical health services, and iii) the health risks of exposure to petrochemicals. This analysis demonstrates how economic exclusion not only affects material wellbeing but also inhibits social participation and integration, contributing to a cycle of disadvantage that perpetuates inequality and injustice.

A strength of this study lies in its detailed exploration of the lived experiences of a group of Ogoni residents from four LGAs, providing insights into the economic impacts of the oil industry from their perspective. A potential limitation of the study, however, is the relatively small sample size (*N* = 22). While the intent was to conduct further interviews, the geographic and logistical challenges and unreliable transportation options that coincided with heightened insecurity around the time of data collection, immediately prior to and after the 2022 Nigerian general elections, together with analysis indicating data saturation, led to the decision to stop the interviews after 22 had been conducted. Despite the challenges, the strong community and industry connections of the first and third author and the research assistant mitigated some of these issues and enabled interviews to be conducted in each of the four LGAs in Ogoniland. This study does not seek to replace or replicate quantitative work on environmental impacts or developmental outcomes. Instead, it aims to bring clarity and detail to how Ogoni residents themselves experience exclusionary impacts and the health and wellbeing consequences of the oil industry. While quantitative data may illustrate important patterns and trends, such as wealth distribution, environmental damage, land use, and employment, qualitative findings provide deeper meaning to these trends through personal stories and experiences.

## Conclusion

This study provides a detailed examination of economic exclusion and its impacts on the health and wellbeing of residents in the Ogoni region. It sought to address an evidence gap by connecting the economic and environmental consequences of oil industry activities to the lived experiences and health outcomes of the Ogoni people. The findings, viewed through the lens of the social exclusion theory, provide insights into the lived experiences of these compounding and systemic injustices for the Ogoni people. Further research should investigate the long-term health impacts of continuous exposure to oil pollution at the population level, and more robustly evaluate the effectiveness of existing remediation efforts. Our study also indicates the need to better trace the psychosocial impacts of economic exclusion, including the effects on mental health, community cohesion, and social capital. Finally, more work is certainly needed understand the gendered dimensions of economic exclusion, examining how women and men experience and cope with economic vulnerabilities differently within oil-impacted communities.

Findings from this study highlight the exclusionary impacts of the oil industry at the intersection of damaged livelihoods and loss of family income, with increased economic vulnerability reducing individuals’ and families’ ability to meet basic needs including healthcare. Meanwhile lack of progress on environmental remediation and non-transparent wealth distribution schemes have exacerbated resident’s experiences of economic displacement and contribute to ongoing exposure to soil, air and water pollution; These findings underscore the importance of strengthening policy interventions that align with international frameworks, such as the Sustainable Development Goals, and Nigerian policy goals which include tackling systemic governance and regulatory challenges, address economic inequalities, and ensuring robust environmental remediation and service infrastructure at both state and LGA levels.

## Supplementary Information

Supplementary Material 1.

## Data Availability

The data gathered for this qualitative investigation is comprised of transcribed interviews with residents in the Ogoni region of the Niger Delta in Rivers State, Nigeria. These interviews were recorded with the participants’ approval and transcribed verbatim. The transcripts were preserved on password-protected computer systems at James Cook University in Townsville, Queensland State, Australia. Confidentiality measures were implemented to safeguard the participants’ privacy, and all personal details were kept anonymous. Access to the information is restricted to the research team members involved in the study, and any dissemination of results will be carried out in a manner that preserves the anonymity of the participants.
