# Supplementary material for: “Economic exclusion and the health and wellbeing impacts of the oil industry in the Niger Delta region: a qualitative study of Ogoni experiences”
Source: Int J Equity Health. 2024 Sep 12;23:183. doi: 10.1186/s12939-024-02248-7 (PMC11391646; doi:10.1186/s12939-024-02248-7)
Supplement: Supplementary file 1 — Supplementary Material 1. [file 12939_2024_2248_MOESM1_ESM.pdf]

Question Guide – Key Questions and Probes

Introduction

- Can you tell me about living in Ogoniland?

- How long have you and your family been here / where is their land / what sort of work do you do?

- Can you describe what life is like for you living in Ogoni?
  - *What are the things in your life that make you feel happiest / most connected?*
  - *Are there any things in your life that make you feel frustrated / angry?*

Key Questions

- Can you tell me what your experiences have been with the oil industry in Ogoni region?
  - *What are the good things (if any)*
  - *What are the bad things (if any)*
  - *From your perspective, who are the big people and organisations involved in oil activity in Ogoniland?*

- In your experience, what have been the biggest changes or impacts in the community linked to oil operations? [Anticipate strong focus on environmental impacts – probe further on following topics]

  - *Can you tell me about how the oil industry has impacted your work and ability to earn an income?*

  - *Can you tell me about how the oil industry has impacted social life or social bonds in your community?*

  - *Can you tell me about how the oil industry has impacted Ogoni cultural life?*

  - *How do you think oil industry has impacted politics in Ogoniland, and more generally?*
    - *Has the oil industry changed what you think about politics and politicians?*

- Can we talk some more about those impacts you have mentioned; do you think any of these challenges have affected your or your family's health and wellbeing?
  - *Tell me more about how [social, cultural, economic and political impacts] have influenced [yours/family] health.*

- In your experience, how have families and communities coped with each of these impacts?
  - *Do you have any examples / stories?*
